# First description of the male of *Draconarius jiangyongensis* (Peng et al., 1996) (Araneae, Agelenidae)

**DOI:** 10.3897/zookeys.601.8349

**Published:** 2016-06-29

**Authors:** Zhuoer Chen, Haiqiang Yin, Xiang Xu

**Affiliations:** 1 College of Life Sciences, Hunan Normal University, Changsha 410081

**Keywords:** China, Hunan, spider, Coelotinae, Coelotes, Langshan Mountain

## Abstract

The male of *Draconarius jiangyongensis* (Peng, Gong & Kim, 1996) is described for the first time from Xinning County, Hunan Province, China. Morphological descriptions and illustrations of both sexes of this species are given in this study. The placement of this species in *Draconarius* is doubted.

## Introduction

The spider genus *Draconarius* Ovtchinnikov, 1999 is distributed in Central and East Asia and has a high level of species diversity with 244 species described to date (World Spider Catalog 2016, Wang 2016). A total of 151 *Draconarius* species has been reported from China, but more than half of these species are described from only the male or female (World Spider Catalog 2016).

*Draconarius jiangyongensis* (Peng, Gong & Kim, 1996) was first described as a member of the genus *Coelotes* Blackwall, 1840 based on six female specimens from Jiangyong County, Hunan Province, China (Peng et al. 1996). Wang (2003) transferred this species to the genus *Draconarius*. *Draconarius jiangyongensis* was illustrated by Song et al. (1999), and redescribed by Wang (2003) and Yin et al. (2012), but only based on the females from the type locality.

During the expedition to Langshan National Geopark in November of 2014, ten females and eleven males were identified to be *Draconarius jiangyongensis* based on comparison with the type specimens. The female is redescribed here and the male is described for the first time in the present study.

## Material and methods

Specimens were examined with an Olympus SZX16 stereomicroscope and an Olympus BX53 compound microscope. Photos were taken with a Canon PowerShot G12 digital camera mounted on an Olympus BX53 compound microscope. Both the male palp and the female epigyne were examined and illustrated after being dissected from the spider bodies. All specimens examined in this study are deposited in the College of Life Sciences, Hunan Normal University (HNU).

All measurements are given in millimeters. Eye diameters are taken at the widest point. Leg measurements are given as: total length (femur, patella + tibia, metatarsus, tarsus). Abbreviations used in the text are as follows:

AME
 anterior median eyes 


ALE
 anterior lateral eyes 


MOA
 median ocular area 


PME
 posterior median eyes 


PLE
 posterior lateral eyes 


## Taxonomy

### Family Agelenidae C. L. Koch, 1837 Genus *Draconarius* Ovtchinnikov, 1999

#### Draconarius jiangyongensis

Taxon classificationAnimaliaAraneaeAgelenidae

(Peng, Gong & Kim, 1996)

Figs 1 , 2 , 3 , 4

Coelotes jiangyongensis Peng et al. 1996: 19, figs 7–9 (description and illustration of ♀); Song et al. 1999: 376, figs 220J–K (♀ figures reproduced from Peng et al. 1996).
Draconarius jiangyongensis : Wang 2003: 536, figs 36A–B, 96B (transferred from Coelotes); Yin et al. 2012: 1010, figs 521a–c (redescription and illustration of ♀).

##### Type material examined.

Holotype, 1♀ (HNU), Jiangyong County, **Hunan Province, China**, 1 October 1991, Liansu Gong leg.; paratypes, 4♀ (HNU), same data as holotype.

##### Additional material examined.

**Hunan Province**, Xining County, Langshan National Geopark: 2♀ (HNU), Tianyixiang (26°21.218'N, 110°48.246'E, 590m), 21.11.2014; 3♀, 2♂ (HNU), same locality as above (26°21.447'N, 110°48.190'E, 560–640m), 22.11.2014; 1♀, 1♂ (HNU), Cave Feiliandong (26°21.447'N, 110°47.921'E, 400m), 23.11.2014; 1♀ (HNU), Bajiaozhai (26°16.354'N, 110°44.308'E, 820m), 24.11.2014; 5♀, 8♂ (HNU), Peak Lajiaofeng (26°23.135'N, 110°48.464'E, 400–640m), 27.11.2014. All specimens are collected by hand picking by Haiqiang Yin, Cheng Wang, Bing Zhou, Jiahui Gan and Yuhui Gong.

##### Diagnosis.

**Figure 1.**
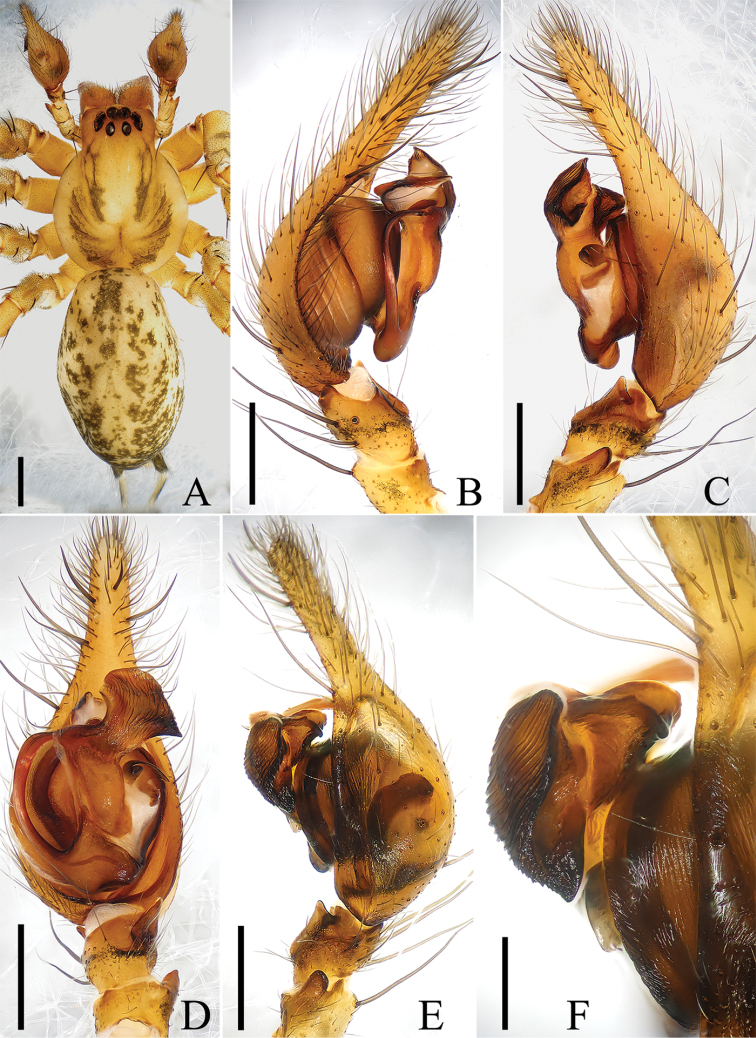
Male of *Draconarius jiangyongensis*. **A** Habitus, dorsal view **B** Left palp, prolateral view **C** Ditto, retrolateral view **D** Ditto, ventral view **E** Ditto (after maceration), retrolateral view **F** Conductor (after maceration), retrolateral view. Scales: **A** = 1 mm; **B**–**D**, **E** = 0.5 mm; **F** = 0.2 mm.

**Figure 2.**
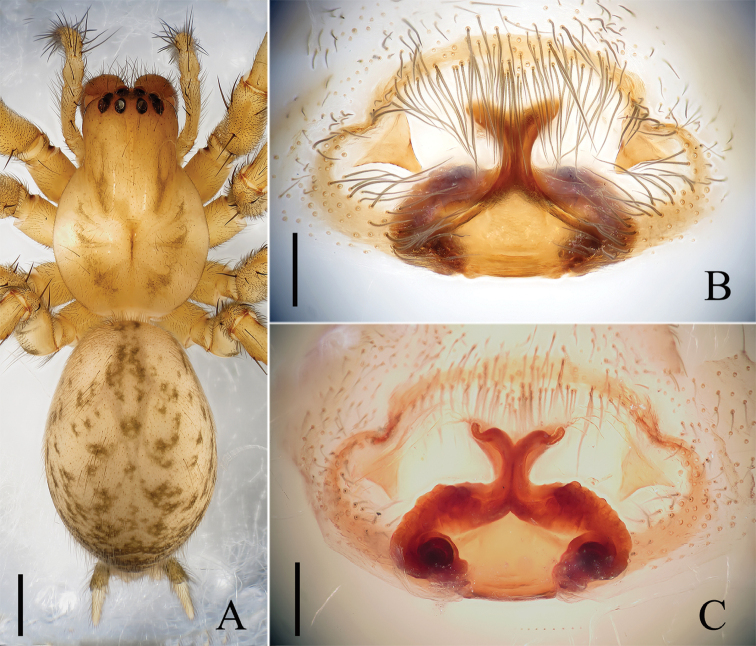
Female of *Draconarius jiangyongensis*. **A** Habitus, dorsal view **B** Epigyne, ventral view **C** Vulva, dorsal view. Scales: **A** = 1 mm; **B**–**C** = 0.2 mm.

**Figure 3.**
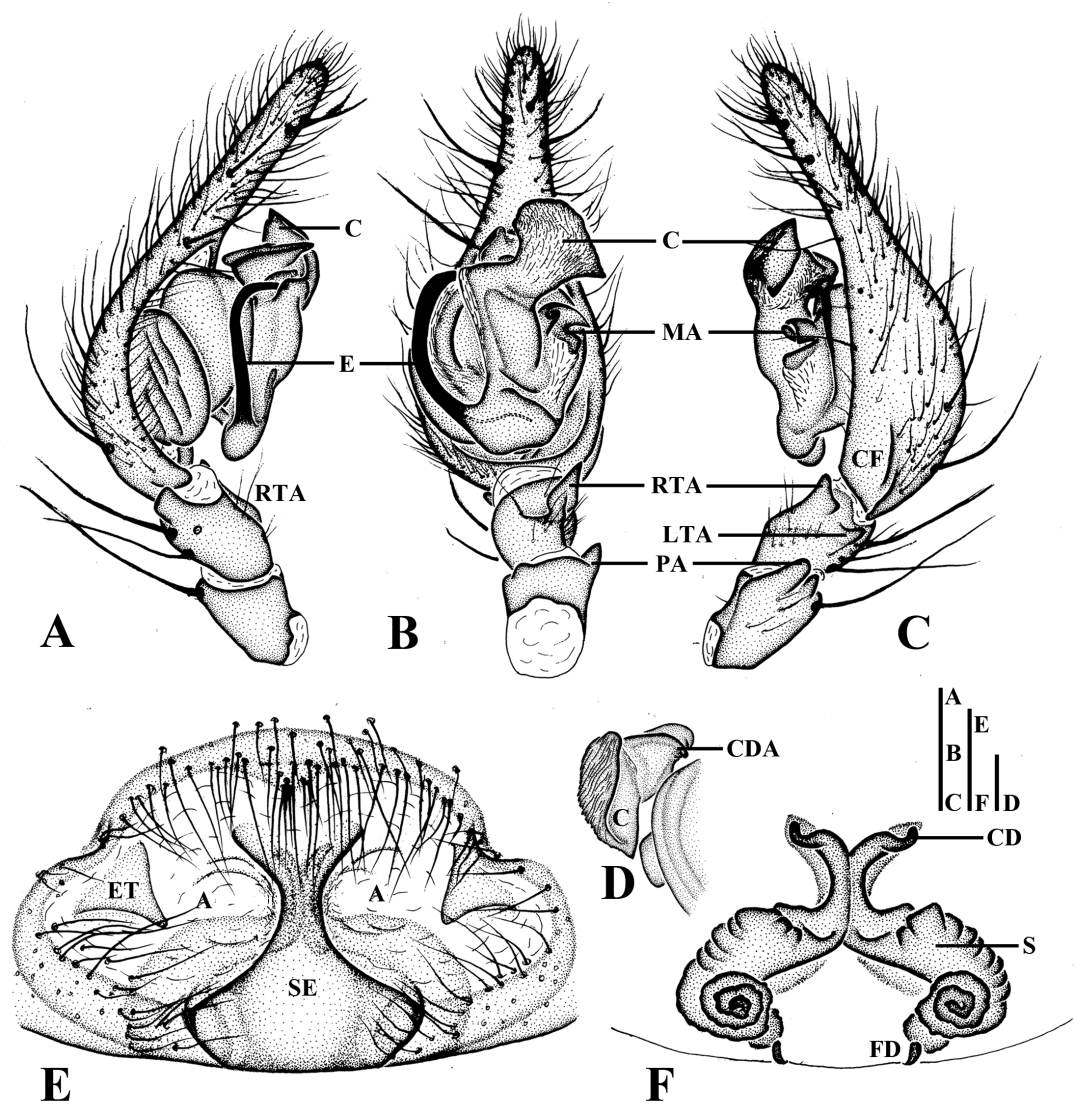
*Draconarius jiangyongensis*. **A–D**: **A** Male left palp, prolateral view **B** Ditto, ventral view **C** Ditto, retrolateral view **D** Conductor (after maceration), retrolateral view **E, F** Female: **E** Epigyne, ventral view **F** Vulva, dorsal view. Abbreviations: A — atrium; C — conductor; CD — copulatory duct; CDA — dorsal conductor apophysis; CF — cymbial furrow; E — embolus; ET — epigynal teeth; FD — fertilization duct; LTA — lateral tibial apophysis; MA — median apophysis; PA — patellar apophysis; RTA — retroventral tibial apophysis; SE — septum; S — spermathecae. Scales: **A**–**C** = 0.5 mm; **D**–**F** = 0.2 mm.

Female of *Draconarius jiangyongensis* can be distinguished from other *Draconarius* by the presence of a vase-shaped septum of epigyne (Figs 2B, 3E), the anteriorly originating and laterally extending copulatory ducts, and the spermathecae widely separated basally and contiguous distally (Figs 2B, C, 3E, F). The male of *Draconarius jiangyongensis* is similar to *Draconarius yadongensis* (Hu & Li, 1987) in having a simple conductor, an embolus arising at approximately 10 o’clock (left palp) and the short cymbial furrow (Figs 1C, D, 3B, C), but can be distinguished from the latter by the shape of the conductor (the conductor axe-shaped, with a wrinkly surface in *Draconarius jiangyongensis*, but narrow with a sharp end tip and broad dorsal edge in *Draconarius yadongensis*) (Figs 1D, F, 3B, D).

##### Description.

**Male.** Total length 8.7. Carapace 4.1 long, 3.1 wide; opisthosoma 4.5 long, 2.9 wide. Clypeus height 0.15. Cephalic part much elevated from the thoracic region. Cervical and radial grooves greyish-black (Fig. 1A). Eye sizes and interdistances: ALE 0.18, AME 0.20, PLE 0.16, PME 0.16; ALE-AME 0.04, AME-AME 0.06, PLE-PME 0.08, PME-PME 0.16; MOA 0.54 long, anterior width 0.58, posterior width 0.70 (Fig. 1A). Labium reddish-brown, 0.6 long, 0.6 wide. Sternum brown, slightly longer than wide (2.3 long, 1.9 wide). Chelicerae with three promarginal and four retromarginal teeth. Leg measurements: I 18.0 (4.5, 6.0, 4.9, 2.6), II 15.8 (4.2, 5.3, 4.2, 2.1), III 14.0 (3.7, 4.3, 4.0, 2.0), IV 17.8 (4.5, 5.7, 5.4, 2.2). Opisthosoma with dorsal pattern composed of several chevrons patterns (Fig. 1A).

Male palp (Figs 1B–F, 3A–D): femur nearly 3/4 length of cymbium; patellar apophysis large; retroventral tibial apophysis moderately long, about 2/3 length of tibia; lateral tibial apophysis small, widely separated from retrolateral tibial apophysis; cymbial furrow short, less than 1/3 length of cymbium; conductor broad, axe-shaped, with a wrinkly surface; dorsal conductor apophysis large; median apophysis large, with a sharp end in ventral view; embolus long and flat, arising at approximately 10 o’clock and encircling for about 180 degrees around bulb.

**Female.** Total length 8.60. Carapace 4.0 long, 2.9 wide; opisthosoma 4.6 long, 3.1 wide. Clypeus height 0.14. Eye sizes and interdistances: ALE 0.2, AME 0.22, PLE 0.18, PME 0.18; ALE-AME 0.04, AME-AME 0.08, PLE-PME 0.10, PME-PME 0.20; MOA 0.52 long, anterior width 0.58, posterior width 0.66. Labium greyish brown, 0.60 long, 0.50 wide. Sternum brown, slightly longer than wide (2.10 long, 1.80 wide). Leg measurements: I 14.3 (3.8, 5.0, 3.5, 2.0), II 12.2 (3.5, 4.2, 3.0, 1.5), III 10.8 (3.0, 3.6, 2.9, 1.3), IV 14.1 (4.0, 4.8, 3.5, 1.8). Promarginal and retromarginal teeth of chelicera and the dorsal pattern of opisthosoma are the same as male (Fig. 2A).

Epigyne (Figs 2B, C, 3E, F): teeth triangular, large and thin, located anterolaterally; septum large, with the base much wider than the stem; atrium divided into two parts by septum; the bases of spermathecae highly convoluted and separated about two times their diameter from each other, and the distal ends of spermathecae contiguous; copulatory ducts short, anteriorly situated and laterally extending.

##### Remark.

The *Draconarius* and *Coelotes* are two most species-rich genera in the Coelotinae, with 244 and 183 species described to date, respectively. Most of those species were described based on only the male or female. As a result, some might be incorrectly placed. This species described here is more likely to be a member of the genus *Coelotes* than *Draconarius* based on the following combination of characters: the large epigynal teeth, the atrium (atrium divided into two parts by septum) and short copulatory ducts in the female; the large patellar apophysis, the short and prolaterally originating embolus and the short cymbial furrow (less than 1/3 length of the cymbium) in the male. It differs from *Coelotes atropos* (Walckenaer, 1830) by the presence of septum. It also differs from the type species and many other species of *Draconarius* (for example, *Draconarius guizhouensis* (Peng, Li & Huang, 2002), *Draconarius latellai* Marusik & Ballarin, 2011 and so on) by the number of cheliceral teeth (this species with three promarginal and four retromarginal teeth while the type species and many other species of *Draconarius* have three promarginal and two retromarginal teeth.)

##### Distribution.

China (Hunan).

**Figure 4.**
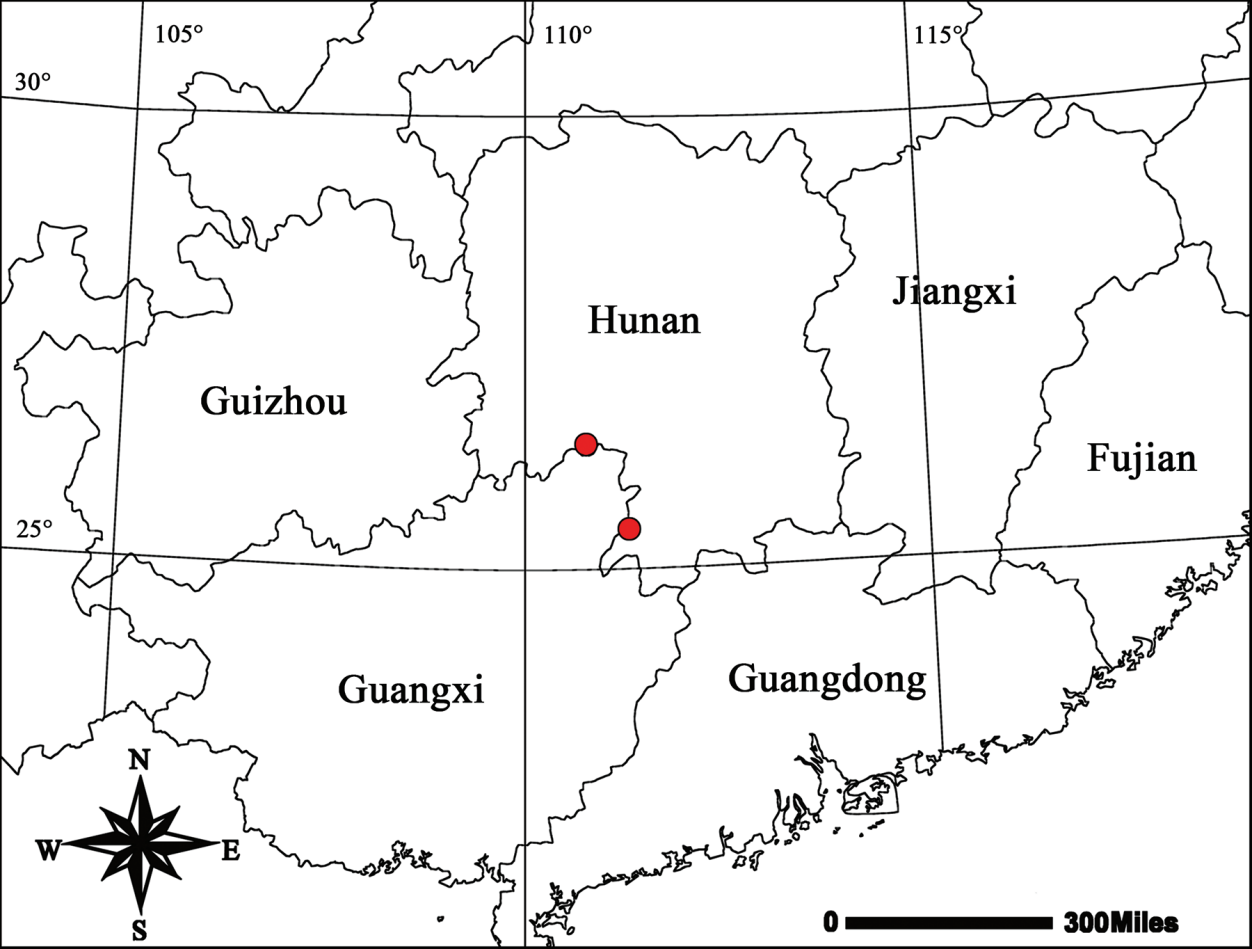
Distribution records of *Draconarius jiangyongensis*.

## Supplementary Material

XML Treatment for Draconarius jiangyongensis
